# Multichannel Signals Reconstruction Based on Tunable *Q*-Factor Wavelet Transform-Morphological Component Analysis and Sparse Bayesian Iteration for Rotating Machines

**DOI:** 10.3390/e20040263

**Published:** 2018-04-10

**Authors:** Qing Li, Wei Hu, Erfei Peng, Steven Y. Liang

**Affiliations:** 1 College of Mechanical Engineering, Donghua University, Shanghai 201620, China; 2 World Transmission Technology (Tianjin) Co., Ltd., Tianjin 300404, China; 3 George W. Woodruff School of Mechanical Engineering, Georgia Institute of Technology, Atlanta, GA 30332-0405, USA

**Keywords:** multichannel signals reconstruction, tunable *Q*-factor wavelet transform-morphological component analysis (TQWT-MCA), sparse Bayesian iteration, redundant step-impulse dictionary, gearbox

## Abstract

High-speed remote transmission and large-capacity data storage are difficult issues in signals acquisition of rotating machines condition monitoring. To address these concerns, a novel multichannel signals reconstruction approach based on tunable *Q*-factor wavelet transform-morphological component analysis (TQWT-MCA) and sparse Bayesian iteration algorithm combined with step-impulse dictionary is proposed under the frame of compressed sensing (CS). To begin with, to prevent the periodical impulses loss and effectively separate periodical impulses from the external noise and additive interference components, the TQWT-MCA method is introduced to divide the raw vibration signal into low-resonance component (LRC, i.e., periodical impulses) and high-resonance component (HRC), thus, the periodical impulses are preserved effectively. Then, according to the amplitude range of generated LRC, the step-impulse dictionary atom is designed to match the physical structure of periodical impulses. Furthermore, the periodical impulses and HRC are reconstructed by the sparse Bayesian iteration combined with step-impulse dictionary, respectively, finally, the final reconstructed raw signals are obtained by adding the LRC and HRC, meanwhile, the fidelity of the final reconstructed signals is tested by the envelop spectrum and error analysis, respectively. In this work, the proposed algorithm is applied to simulated signal and engineering multichannel signals of a gearbox with multiple faults. Experimental results demonstrate that the proposed approach significantly improves the reconstructive accuracy compared with the state-of-the-art methods such as non-convex Lq (q = 0.5) regularization, spatiotemporal sparse Bayesian learning (SSBL) and L1-norm, etc. Additionally, the processing time, i.e., speed of storage and transmission has increased dramatically, more importantly, the fault characteristics of the gearbox with multiple faults are detected and saved, i.e., the bearing outer race fault frequency at 170.7 Hz and its harmonics at 341.3 Hz, ball fault frequency at 7.344 Hz and its harmonics at 15.0 Hz, and the gear fault frequency at 23.36 Hz and its harmonics at 47.42 Hz are identified in the envelope spectrum.

## 1. Introduction

Rotating machines, as key mechanical components, have been widely used in modern industries, they often experience severe multi-mode vibrations when exposing to extremely harsh operation environment such as high-temperature, high humidity and chemical corrosion, etc., those vibrations may cause malfunctions, failure and will significantly reduce the fatigue life or even result in catastrophic accidents. Therefore, it is urgent to timely detect/diagnosis the operating condition of rotating machines, and ultimately predict its durability and remaining useful life (RUL) to ensure the equipment runs effectively. However, prognostic and health management (PHM) is a perennial/long-term concern, an intractable issue, data storage and remote transmission, which comes up in PHM, brings more pressure on monitoring with data increasing daily [1,2,3]. 

Currently, remote transmission and data storage in engineering applications suffer from the drawbacks of low speed and low capacity, the fidelity of the signal is also difficult to guarantee, and traditional hardware and memory cannot meet industrial needs. Compressed sensing (CS) [4,5] is a new framework in signal acquisition which collects the sample data and compress those data simultaneously, and then the compressed signals are sent to a remote terminal through the Internet and Bluetooth, at the terminal, the original signal can be recovered without compromising on the reconstruction quality, which reduces the collecting period and the level of requirement on hardware, and has wide applications, such as biomedical imaging, optical/microwave imaging, Earth remote sensing, biological computing, and other fields [6,7,8,9,10]. 

The core purpose of CS framework is to efficiently recover the raw signal with high accuracy from the compressed data, thus, a variety of methodologies have been proposed in recent years. Roughly, the existing construction algorithms can be divided into four classes:

(1) greedy pursuit algorithms such as matching pursuit (MP) [11], orthogonal matching pursuit (OMP) [12,13], etc.;
(2) convex regularization methods such as the family of L1-norm [14];
(3) non-convex regularization methods such as the family of nonconvex Lp-norm (0 < p < 1) [15,16]; and
(4) sparse low rank matrix (SLRM) approaches such as non-separable SLRM regularization [17,18,19], etc.

Those CS algorithms and their optimized algorithms have achieved successful applications in industrial applications, including some applications on mechanical fault diagnosis and condition monitoring. As a matter of fact, unlike the images acquisition which collect the image data one by one, the vibration signals acquisition system aims at sampling the data from different location with multiple channels (e.g., eight channels, 16 channels). Thus, the traditional CS algorithm is designed for recovering single-image or single-channel signal, when recovering multichannel signals, the CS has to recover the signals channel by channel, which is time consuming and may not be suitable for real-time condition monitoring of mechanical equipment with multichannel signals. 

Additionally, for many multichannel vibration signals, such as bearing or gearbox failures, there is strong spatiotemporal relationships among the signals from different channels, for example, the signals collected from X/Y directions at same location, the shaft centerline orbit (SCO) calculated by both directions could be used for misalignment and eccentric testing of the bearing or gearbox, unfortunately, traditional CS algorithm ignores it, which means the spatiotemporal relationships are not considered. Another aspect should be also highlighted, it is computing time. Generally, the computing time of an algorithm required for a solution greatly depends on the dimension and structure of multi-channel data, thus exploiting the inter-channel correlation and dealing with large-scale signal reconstruction in real-time is necessary and very beneficial for PHM of rotating machines.

For the issues of spatiotemporal relationship, the researchers focused on sparse Bayesian learning methods. In [20,21], Zhang et al. developed a framework of block sparse Bayesian learning (BSBL) for electroencephalography (EEG) signal reconstruction in terms of the multiple measurement vectors (MMV) problem. Furthermore, in order to exploit the temporal and spatial correlation structure of an EEG signal, Zhang et al. [22] proposed a spatiotemporal sparse Bayesian learning (SSBL) for EEG signal reconstruction analyzing its stability based on the compression ratio. However, it is noted that the above proposed sparse Bayesian learning methods for compressive sensing made a critical assumption that the dictionary atoms, such as discrete wavelet transform (DWT) or discrete cosine transform (DCT), are used without any matching in the EEG signal implementation. As is well known, no matter what the physical structure of the signal, if the compressed signal is not sparse, in practical engineering, the dictionary atom matching inevitably occurs in the CS, which will actually affect the dynamic behavior and may lead to the oscillation phenomenon or attenuation of the signal. 

More importantly, due to the periodic impulses caused by the localized fault in rotating machines, usually considered as the low-resonance component (LRC), which are key information for condition monitoring of rotating machines and hidden in natural modulated components and additive background noise [23,24,25,26,27], if the SBL method recovers the raw data without any preprocessing, the LRC may distortion and alias with high-resonance components, resulting in the loss of the fault characteristic frequencies. Unfortunately, the conventional SBL approaches treat all vibration signal amplitudes equally, thus, ignore a fact that the LRC may contain more useful information of periodical impulses and should be preserved with a larger coefficient. When the LRC are reconstructed failure, which would lead to the misdiagnosis in the terminal. 

In this paper, aiming at the issue of recovering the multichannel signals from their original observation, a novel reconstruction approach based on TQWT-morphological component analysis (TQWT-MCA), sparse Bayesian iteration combined with step-impulse dictionary is proposed, using the eight-channel vibration signals of a gearbox with multiple faults as a research object. To begin with, the raw signal is decomposed into LRC and HRC by the TQWT-MCA method, the dictionary atom is designed to match the physical structure of generated LRC impulses, then, both LRC and HRC are reconstructed by the sparse Bayesian iteration algorithm. Meanwhile, the time-frequency and envelope spectrum analysis are implemented to test the fidelity-degree of the reconstructed components. Finally, the proposed method is validated via eight-channel signals of the gearbox dataset collected in practical engineering, the reconstruction and the diagnosis results are superior to the other state-of-the-art methods, such as convex L1-norm, OMP, or non-convex Lp-norm techniques, etc.

The main contributions of this paper are summarized as follows:

(1) compared to the single-channel signal, the reconstruction of multichannel signals is addressed by the proposed TQWT-MCA and sparse Bayesian iteration method. Meanwhile, the issue of time consumption is improved significantly.
(2) the spatiotemporal relationships among the signals from different channels are considered via the sparse Bayesian iteration algorithm.
(3) the dictionary atom is designed to match the physical structure of periodic impulses caused by the localized fault, thus, the signal distortion problem is addressed effectively.
(4) the periodical impulses-loss problem is addressed via a pre-processing method, i.e., TQWT-MCA technique, in this paper, the periodical impulses can be separated accurately from the external noise and interference components, which means that the periodical impulses and their fault frequencies will be saved.

The layout of the paper is organized as follows: In Section 2, the TQWT-morphological component analysis (TQWT-MCA) framework is presented. Section 3 describes the sparse Bayesian iteration approach and flow chart of the proposed method in detail. Verification of the methodology as applied to the simulated vibration signal is provided in Section 4. Engineering application results are presented in Section 5. Finally, discussions and conclusions are drawn in Section 6.

## 2. TQWT-MCA Algorithm

### 2.1. Tunable Q-Factor Wavelet Transform

The TQWT is a flexible discrete wavelet transform for oscillatory signal processing so that the Q-factor of the wavelet is easily tuned and continuously adjustable [28]. The TQWT consists of two iterative band-pass filter banks, i.e., the high resonance component filter and the low resonant component filter. The resonance characteristics of oscillatory signal can be represented by quality factor *Q*, the *Q*-factor of a band-pass filter is the ratio of its center frequency to its bandwidth, i.e., $Q=f_{c}/B_{w}$, in which $B_{w}$ is bandwidth of signal and $f_{c}$ denotes center frequency. The main changeable parameters of the TQWT are quality factor-*Q*, redundancy rate *r*, and the number of decomposition scales/levels *j*.

**Figure 1:**
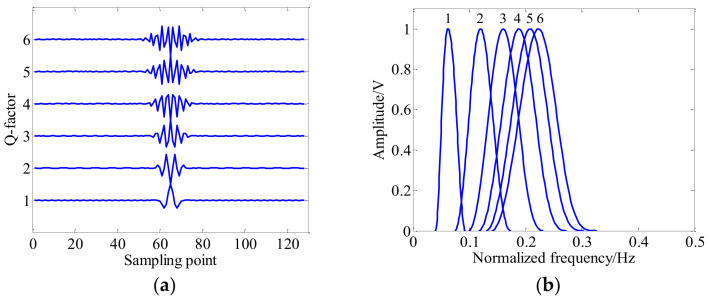
Wavelet waveform and frequency responses with fixed *j* scale and different *Q*-factors (e.g., *j* = 2, *Q* = 1, 2, 3, 4, 5, 6). (**a**) Wavelet tome domain waveform; and (**b**) frequency responses.

**Figure 2:**
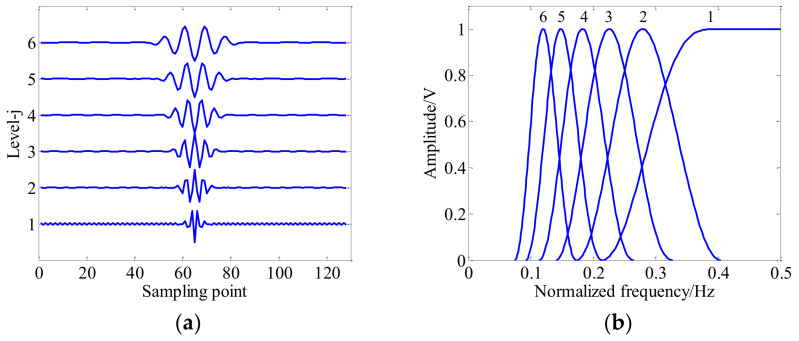
Wavelet waveform and frequency responses with fixed *Q*-factor and different *j* scales (e.g., *Q* = 2.5, *j* = 1, 2, 3, 4, 5, 6). (**a**) Wavelet time domain waveform; and (**b**) frequency responses.

Commonly, the factor-*Q* measures the oscillatory behavior and waveform shape of wavelet waveform, and the decomposition level *j* controls the expansion extent and bandpass location of wavelet waveform. Figure 1 illustrates the wavelet waveform and frequency response curves with different a fixed scale (i.e., *j* = 2) and different *Q*-factors (e.g., *j* = 2, *Q* = 1, 2, 3, 4, 5, 6). As shown in Figure 1, the wavelet waveform becomes more oscillatory with the increase of factor-*Q*. Figure 2 shows the wavelet time-domain waveform and frequency response curves with a fixed *Q*-factors (i.e., *Q* = 2.5) and different *j* scales (e.g., *j* = 1, 2, 3, 4, 5, 6). It can be observed in the Figure 2 that the wavelet waveform of the high scale (e.g., *j* = 6) is wider than the low scale (e.g., *j* = 2). Generally, the redundancy rate *r* = 3 has been recommended in [28].

For every level of TQWT decomposition, the input signal s(n) with sampling frequency *f*s can decomposed into sub-band c_0_[n] and sub-band d_1_[n], where c_0_[n] and d_1_[n] are low-pass and high-pass sub-band signals with sampling frequencies *αf*s and *βf*s, respectively, and parameters *α* and *β* are scaling factors. Furthermore, the low-pass filter *F*_0_(*ω*) and low-pass scaling α*f*s is applied to generated c_0_[n] and the low-pass filter *F*_1_(*ω*) and low-pass scaling *βf*s is used to obtain d_1_[n]. However, to prevent excessive redundancy and achieve perfect reconstruction, the scaling parameters should obey the following principle, i.e., 0 < *α* < 1; 0 < *β* ≤ 1 and *α* + *β* > 1. Mathematically, the low-pass *F*_0_(*ω*) and high-pass filter *F*_1_(*ω*) are given as follows:

(1) $$F_{0}(\omega )=\left\{ \begin{array}{ll} 1, & \left|\omega \right|<(1-\beta )\pi \\ \theta (\frac{\omega +(\beta -1)\pi }{\alpha +\beta -1}), & (1-\beta )\leq \left|\omega \right|<\alpha \pi \\ 0, & \alpha \pi \leq \left|\omega \right|\leq \pi \end{array}\right.$$

(2) $$F_{1}(\omega )=\left\{ \begin{array}{ll} 0, & \left|\omega \right|<(1-\beta )\pi \\ \theta (\frac{\alpha \pi -\omega }{\alpha +\beta -1}), & (1-\beta )\leq \left|\omega \right|<\alpha \pi \\ 1, & \alpha \pi \leq \left|\omega \right|\leq \pi \end{array}\right.$$

It is to be noted that θ(ω) is the frequency response of Daubechies filter that have two vanishing moments. The θ(ω) is defined with the following expression:

(3) $$\theta (\omega )=0.5\times (1+\cos(\omega ))\times \sqrt{2-\cos(\omega )},\left|\omega \right|\leq \pi$$

The *Q*-factor *Q* and redundancy rate *r* can be expressed in terms of parameters α and β as follows, i.e.:

(4) $$Q=\frac{f_{c}}{B_{w}}=\frac{2-\beta }{\beta },r=\frac{\beta }{1-\alpha }$$

where *fc* and *B_w_* are center frequency and bandwidth of the frequency response of sub-band signal.

### 2.2. TQWT-Morphological Component Analysis

Given an observed signal $x=x_{1}+x_{2}$, with $x\in {\mathbb{R}}^{N}$, $x_{1}\in {\mathbb{R}}^{N}$ denotes the low-resonance/frequency signal and $x_{2}\in {\mathbb{R}}^{N}$ denotes the high-resonance/frequency signal. The objective of the morphological component analysis (MCA) is to separate signal *x*_1_ and signal *x*_2_ individually. Meanwhile, assuming that signal *x*_1_ and signal *x*_2_ can be sparsely represented via transform bases *s*_1_ and *s*_2_, respectively. Hence, the separation problems can be solved by minimization L1-norm approach, i.e.:

(5) $$F(w_{1},w_{2})={\left\|x-s_{1}w_{1}-s_{2}w_{2}\right\|}_{2}^{2}+\lambda_{1}{\left\|w_{1}\right\|}_{1}+\lambda_{2}{\left\|w_{2}\right\|}_{1}$$

where $\lambda_{1}$ and $\lambda_{2}$ are regularization parameters. Then, the signal *x*_1_ and signal *x*_2_ could be approximately estimated with:

(6) $$\overset{\wedge }{x_{1}}=s_{1}w_{1}\mathrm{and}\overset{\wedge }{x_{2}}=s_{2}w_{2}$$

It is important that the two utilized transform bases, *s*_1_ and *s*_2_, have a low mutual coherence, that is, the transform base *s*_1_ and transform base *s*_2_ have minimal correlation, so that the signal *x*_1_ and signal *x*_2_ can be decomposed successfully, in this work, the high-*Q* and low-*Q* factors are utilized for signal decomposition based on MCA.

Commonly, the high-resonance signal can be efficiently represented with a high-*Q* factor and likewise the low-resonance signal can be efficiently represented with a low-*Q* factor. Moreover, the high-*Q* factor should be designed so that it is sufficiently higher than the low-*Q* factor to satisfy the oscillation behavior, however, if the high-*Q* factor is too high, the estimated signal may not be well matched to the oscillatory behavior of high-resonance signal, accordingly degrading the results of MCA, and this is also true for too low *Q* factor. Therefore, the key of the TQWT-MCA is to select the appropriate *Q* factors so as to roughly reflect the oscillatory behavior of the two sub-signals *x*_1_ and signal *x*_2_. 

The method given in [29] to suggest us to estimate the appropriate *Q* factors based on the following maximum inner product criterion (MIPC), which is:

(7) $$\rho (f_{1},f_{2})=\left\{ \begin{array}{ll} 0, & f_{2}\leq f_{1}(\frac{1-1/\left(2Q_{1}\right)}{1+1/\left(2Q_{2}\right)})\\ \sqrt{\frac{Q_{1}Q_{2}}{f_{1}f_{2}}}[f_{2}(1+\frac{1}{2Q_{2}})-f_{1}(1-\frac{1}{2Q_{2}})], & f_{1}(\frac{2-1/Q_{1}}{2+1/Q_{2}})\leq f_{2}\leq f_{1}(\frac{2-1/Q_{1}}{2-1/Q_{2}})\\ \sqrt{\frac{f_{2}Q_{1}}{f_{1}Q_{2}}}, & f_{1}(\frac{2-1/Q_{1}}{2-1/Q_{2}})\leq f_{2}\leq f_{1}(\frac{2+1/Q_{1}}{2+1/Q_{2}})\\ \sqrt{\frac{Q_{1}Q_{2}}{f_{1}f_{2}}}[f_{1}(1+\frac{1}{2Q_{2}})-f_{2}(1-\frac{1}{2Q_{2}})], & f_{1}(\frac{2+1/Q_{1}}{2+1/Q_{2}})\leq f_{2}\leq f_{1}(\frac{2+1/Q_{1}}{2-1/Q_{2}})\\ 0, & f_{1}(\frac{2+1/Q_{1}}{2-1/Q_{2}})\leq f_{2} \end{array}\right.$$

where $\rho (f_{1},f_{2})$ is the inner product of *f*_1_ and *f*_2_, *Q*_2_ is defined as the high-*Q* factor and *Q*_1_ is defined as the low-*Q* factor, and *f*_1_ and *f*_2_ are the center frequency of the wavelet transforms of signal *x*_1_ and signal *x*_2_, respectively. The maximum inner product can be written as:

(8) $$\underset{f_{1},f_{2}}{\max}\;\rho (f_{1},f_{2})=\rho_{\max}(Q_{1},Q_{2})=\sqrt{\frac{Q_{1}+1/2}{Q_{2}+1/2}},Q_{2}>Q_{1}$$

If and only if $f_{2}=f_{1}(2+1/Q_{1})(2+1/Q_{2})$. Here, the *Q*_2_ factor should be designed so that it is sufficiently higher than the *Q*_1_ factor, if the *Q*_2_ factor slightly higher than or equal to *Q*_1_ factor, then the maximum inner product is near 1, and the results of component $\overset{\wedge }{x_{1}}$ and $\overset{\wedge }{x_{2}}$ maybe similar to original signal *x*. 

## 3. Signal Reconstruction Based on a Sparse Bayesian Iteration Algorithm

### 3.1. Review of Sparse Bayesian Iteration Framework

The sparse framework is described as follows:

(9) Y=ΦX+V

where the compressed signal $Y\in {\mathbb{R}}^{N\times L}$, $\Phi \in {\mathbb{R}}^{N\times M},N<<M$ is designed measurement matrix, and $X\in {\mathbb{R}}^{M\times L}$ and $V\in {\mathbb{R}}^{N\times L}$ are unknown additive noise. If *L* = 1, the above model is a single measurement vector (SMV), and if *L* > 1, the above model is a multiple measurement vector (MMV). In this algorithm, the purpose is to estimate the signal *X* at the terminal, the original signal *X* is recovered by a CS algorithm, namely:

(10) $$\overset{\wedge }{X}=\arg\;\underset{X}{\min}\;\left\|Y-\Phi X\right\|+\lambda f(X)$$

where λ is a regularization parameter, and *f*(*X*) is a penalty function of *X*, commonly, the penalty function may be L1-norm based penalty, i.e., $f(X)={\left\|X\right\|}_{1}$. If the signal is not sparse, one can seek a dictionary matrix ***D*** such that *X* can be sparsely represented under the dictionary matrix, i.e., X=DZ, where *Z* is the sparse coefficients. Thus, the original signal *X* can be recovered according to:

(11) $$\overset{\wedge }{X}=\arg\;\underset{Z}{\min}\;\left\|Y-\Phi DZ\right\|+\lambda f(Z)$$

where ***D*** is a dictionary matrix, the design of the dictionary atom is presented in Section 3.4. Let us define $X_{\cdot \;l}$ as the *l*-th column of *X*, which is the *l*-th channel of the original vibration signal. Similarly, $Y_{\cdot \;l}$ is the corresponding compressed signal at the *l*-th column. The signal *X* can be viewed as a concatenation of *g* blocks, i.e.:

(12) $$X=[\underset{X_{[1]\;\cdot }}{\underset{︸}{X_{1},X_{2},\cdot \cdot \cdot ,X_{d_{1}}}},\cdot \cdot \cdot ,\underset{X_{[g]\;\cdot }}{\underset{︸}{X_{d_{g-1}+1},X_{d_{g-1}+2},\cdot \cdot \cdot ,X_{d_{g}}}}{]}^{T}={\left[X_{[1]\cdot },X_{[2]\cdot },\cdot \cdot \cdot ,X_{[g]\cdot }\right]}^{T}$$

where $X_{[i]\;\cdot }\in {\mathbb{R}}^{d_{i}\times L}$ is the *i*-th block of signal *X*, and also $\sum_{i=1}^{g}d_{i}=M$. The $\text{\{}d_{1},\cdots ,d_{g}\text{\}}$ is called the block partition. Among the *g* blocks, only *k* (*k* << *g*) blocks are nonzero, but their locations are unknown. In this framework, each block $X_{[i]\;\cdot }\in {\mathbb{R}}^{d_{i}\times L},\;\mathrm{for}\;\forall i$, which satisfy a parameterized Gaussian distribution:

(13) $$P(\mathrm{vec}(X_{\left[i\right]}^{T};\gamma_{i},B,A_{i}))=N(0,(\gamma_{i}A_{i})⊗B)$$

where $B\in {\mathbb{R}}^{L\times L}$ is a matrix that used for capturing the correlation structure of each row of $X_{[i]\;\cdot }$, and $A_{i}\in {\mathbb{R}}^{d_{i}\times d_{i}}$ is a matrix that used for capturing the correlation structure of each column of $X_{[i]\;\cdot }$, the parameter $\gamma_{i}$ is a positive scalar, ⊗ is the symbol of the matrix product. Under the assumption that blocks ${\left\{X_{[i]\;\cdot }\right\}}_{i=1}^{g}$ are mutually uncorrelated, the prior of *X* is:

(14) $$P(\mathrm{vec}(X^{T});B,{\text{\{}\gamma_{i},A_{i}\text{\}}}_{i})=N(0,\Pi ⊗B)$$

where Π is block diagonal matrix defined by:

(15) $$\Pi =\left[\begin{array}{llll} \gamma_{1}A_{1} & & & \\ & \gamma_{1}A_{1} & & \\ & & \ldots & \\ & & & \gamma_{g}A_{g} \end{array}\right]$$

Similarly, the noise vector *V* satisfies:

(16) $$P(V_{i};\lambda ,B)=N(0,\lambda B)$$

Under the assumption that the noises are mutually uncorrelated, the prior of *V* is:

(17) $$P(\mathrm{vec}(V^{T});\lambda ,B)=N(0,\lambda I⊗B)$$

Therefore, the posterior of *X* is given by:

(18) $$P(X_{\cdot \;i}|Y_{\cdot \;i};\lambda ,\Pi )=N(\mu_{\cdot \;i},\sum ),\;\mathrm{for}\;\forall i$$

where the mean $\mu_{\cdot \;i}$ and the covariance matrix ∑ are given by:

(19) $$\mu_{\cdot \;i}=\Pi \Phi^{T}{\left(\lambda I+\Phi \Pi \Phi^{T}\right)}^{-1}Y_{\cdot \;i},\;\mathrm{for}\;\forall i$$

(20) $$\sum ={\left(\Pi^{-1}+\frac{1}{\lambda }\Phi^{T}\Phi \right)}^{-1}=\Pi -\Pi \Phi^{T}{\left(\lambda I+\Phi \Pi \Phi^{T}\right)}^{-1}\Phi \Pi$$

Thus, once the parameters Π and λ are estimated, the maximum posteriori estimate of *X* can be given by the mean of the posterior, i.e.:

(21) $$X=\Pi \Phi^{T}{\left(\lambda I+\Phi \Pi \Phi^{T}\right)}^{-1}Y$$

### 3.2. Iteration Rule for Matrix A

In this work, the parameter Π and λ are estimated by the expectation maximization (EM) method [30,31,32]. Based on the EM method, the *Q*-function for estimating $\left\{\gamma_{i}\right\}$ and $\left\{A_{i}\right\}$ is given by:

(22) $$\begin{array}{l} Q(\Pi )=E_{X|Y;\Theta^{\left(old\right)}}[\log P(X;{\left\{\gamma_{i}\right\}}_{i},{\left\{A_{i}\right\}}_{i})]\\ =-\frac{L}{2}\log\left|\Pi \right|-\frac{1}{2}\sum_{i=1}^{L}E_{X|Y;\Theta^{\left(old\right)}}[X_{\cdot \;i}^{T}\Pi^{-1}X_{\cdot \;i}]\\ =-\frac{L}{2}\sum_{i=1}^{g}\log\left|\gamma_{i}A_{i}\right|-\frac{1}{2}\sum_{l=1}^{L}Tr[\Pi^{-1}(\sum +\mu_{\cdot \;l}\mu_{\cdot \;l}^{T})]\\ =-\frac{L}{2}\sum_{i=1}^{g}d_{i}\log\gamma_{i}-\frac{L}{2}\sum_{i=1}^{g}\log\left|A_{i}\right|-\frac{1}{2}\sum_{l=1}^{L}\sum_{j=1}^{g}\frac{1}{\gamma_{j}}Tr[A_{j}^{-1}(\sum_{\left[j\right]}+\mu_{[j]\;l}\mu_{[j]\;l}^{T})] \end{array}$$

where the $\Theta^{\left(old\right)}$ represents all the parameters estimated in the previous iteration, i.e., $\Theta^{\left(old\right)}=\{\lambda ,\{\gamma_{i},A_{i}\},B\}$, $\sum_{\left[j\right]}$ is the *j*-th diagonal block in the ∑, $\mu_{[j]\;l}$ is the *j*-th block in the *l*-th column of μ, and Tr(⋅) is trace of the matrix. Setting the partial derivative of Equation (22) over $\gamma_{i}$ to zero, we have:

(23) $$\gamma_{i}=\frac{1}{Ld_{i}}\sum_{l=1}^{L}Tr[A_{i}^{-1}(\sum_{\left[i\right]}+\mu_{[i]\;l}\mu_{[i]\;l}^{T})]$$

Setting the partial derivative of Equation (22) over *A_i_* to zero, we have:

(24) $$A_{i}=\frac{1}{L}\sum_{l=1}^{L}\frac{\sum_{\left[i\right]}+\mu_{[i]\;l}\mu_{[i]\;l}^{T}}{\gamma_{i}}$$

To estimate λ, the *Q*-function is given by:

(25) $$\begin{array}{l} Q(\lambda )=E_{X|Y;\Theta^{\left(old\right)}}[\log P(Y|X;\lambda )]\\ =-\frac{NL}{2}\log\lambda -\frac{1}{2\lambda }E_{X|Y;\Theta^{\left(old\right)}}[\sum_{l=1}^{L}{\left\|Y_{\cdot \;i}-\Phi X_{\cdot \;i}\right\|}_{2}^{2}]\\ =-\frac{NL}{2}\log\lambda -\frac{1}{2\lambda }\sum_{l=1}^{L}\left[{\left\|Y_{\cdot \;i}-\Phi \mu_{\cdot \;i}\right\|}_{2}^{2}+E_{X|Y;\Theta^{\left(old\right)}}[{\left\|\Phi X_{\cdot \;i}-\mu_{\cdot \;i}\right\|}_{2}^{2}]\right]\\ =-\frac{NL}{2}\log\lambda -\frac{1}{2\lambda }{\left\|Y_{\cdot \;i}-\Phi \mu \right\|}_{ℱ}^{2}-\frac{1}{2\lambda }Tr(\sum \Phi^{T}\Phi ) \end{array}$$

Setting its derivative over λ to zero, we have:

(26) $$\lambda =\frac{1}{NL}{\left\|Y-\Phi \mu \right\|}_{ℱ}^{2}+\frac{1}{N}Tr(\sum \Phi^{T}\Phi )$$

Thus, the updating rule of λ the parameter is given by:

(27) $$\lambda =\frac{1}{NL}{\left\|Y-\Phi \mu \right\|}_{ℱ}^{2}+\frac{1}{N}\sum_{i=1}^{g}Tr(\sum_{\left[i\right]}\Phi_{\cdot \;[i]}^{T}\Phi_{\cdot \;[i]})$$

where $\Phi_{\cdot \;[i]}$ denotes the *i*-th columns of Φ. Generally, for the noiseless situations, the value of λ is typically set to the a sufficiently small values, such as $\lambda =10^{-5}$, instead of the updating rule Equation (27). In the next section, the matrix ***B*** can be estimated and discussed below. 

### 3.3. Iteration Rule for Matrix B

Assuming signal *X*, $\left\{\gamma_{i}\right\}$ and $\left\{A_{i}\right\}$ have been obtained, following the approach used to derive the temporally-correlated sparse Bayesian learning (T-SBL) algorithm [20,21], the updating rule of the matrix ***B*** is displayed as follows:

(28) $$B=\sum_{i=1}^{g}\gamma_{i}^{-1}X_{[i]\;\cdot }^{T}A_{i}^{-1}X_{[i]\;\cdot }+\lambda^{-1}{\left(Y-\Phi X\right)}^{T}(Y-\Phi X)$$

and:

(29) $$B=\frac{B}{{\left\|B\right\|}_{ℱ}}$$

where $X_{[i]\;\cdot }$ is the *i*-th block in *X*, and the second term in Equation (28) is noise-related. For the noiseless situations, the second term in Equation (28) could be removed or set to the a sufficiently small values, such as $\lambda =10^{-5}$. 

### 3.4. Redundant Dictionary Atom Based on Step-Impulse Equation

In order to guarantee the dictionary atom can match the natural structure of periodical impulses (i.e., LRC) caused by the localized fault, and effectively address the signal distortion problem, the impulse-step-like impact dictionary atom is defined as follows:

(30) $$d=\eta_{1}\cdot a\cdot d_{imp}+\eta_{2}\cdot d_{step}$$

where parameter *a* is the peak value ratio of impulse-like to the step-like impact, $d_{imp}$ is the single degree of freedom impulse-like impact, and $d_{step}$ is the single degree of freedom step-like impact. $\eta_{1}$ and $\eta_{2}$ are adjusting parameters, which are used for adjusting the amplitude of dictionary atom consistent with the amplitude of LRC. The two impacts are defined respectively as follows:

(31) $$d_{imp}=\exp(\frac{-(t-u)}{\tau })\sin(2\pi f_{n}t)$$

(32) $$d_{\mathrm{step}}=\exp(\frac{-(t-u-\Delta t)}{3\tau })\times (-\cos(2\pi \frac{f_{n}}{6}t))+\exp(\frac{-(t-u)}{5\tau })$$

where *f_n_* is natural frequency of system, parameter *τ* is system damping, *u* the time when the impulse-like impact occurs, Δt is the period time that the contact part, such as a gear tooth or bearing ball entering and then exiting from the fault region (e.g., pitting or crack). The detailed formulas and computation steps for Δt are given in our previous work [16]. 

In this paper, the procedures of proposed technique for multichannel signals reconstruction of rotating machinery can be divided into six steps:

(1) Collect the multichannel raw vibration data of rotating machinery using acceleration sensors;
(2) Chose the appropriate parameters, such as, high-factor *Q*_2_ and low-factor *Q*_1_ and regularization parameter $\lambda_{i}$, etc., according to maximum inner product criterion (MIPC) in Equations (7) and (8). The high-resonance component and low-resonance component can be obtained by TQWT-MCA method;
(3) Establish the redundant dictionary atom based on step-impulse equation in Equations (30)–(32), and then apply the sparse Bayesian iteration to respectively reconstruct the high-resonance component (HRC) and low-resonance component (LRC);
(4) Combined high-resonance component and low-resonance component and obtain the final reconstructed signal;
(5) Detect failure frequency and its harmonics based on the final reconstructed signal;
(6) Comparative analysis with other start-of-the art methods.

**Figure 3:**
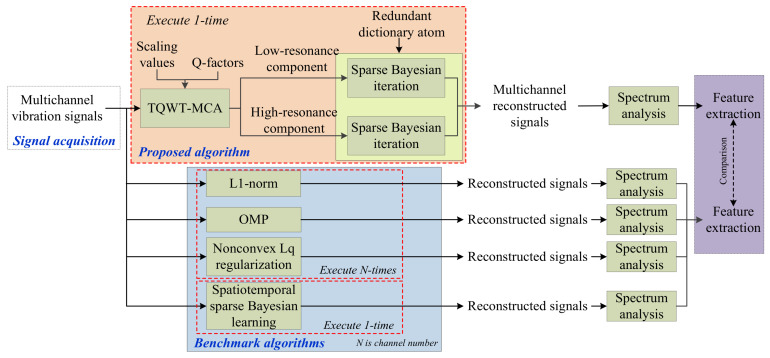
The flow chart of the proposed method for vibration signal reconstruction of rotating machines.

The flow chart of the proposed method for multichannel vibration signal reconstruction of rotating machinery is illustrated in Figure 3. 

## 4. Numerical Simulation Case

**Figure 4:**
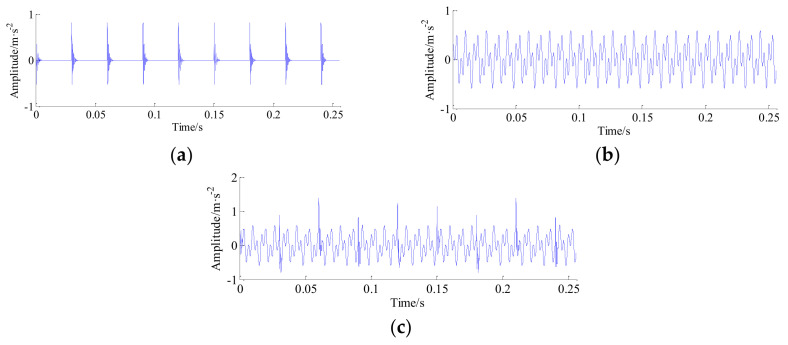
The simulated signal. (**a**) The simulated periodic impulses; (**b**) the simulated high-frequency signal; and (**c**) the simulated synthetic signal.

A numerical simulation is utilized to investigate the effectiveness of the proposed approach for vibration signals reconstruction. In view of the physical structure of vibration signals, here, a low frequency signal is developed to simulate the periodic impulses that are caused by the localized fault, and high frequency signal is designed to simulate the natural modulated signal due to systematic components. The synthetic response function can be described by the following formula:

(33) $$\left\{ \begin{array}{l} x(t)=x_{1}(t)+x_{2}(t)\\ x_{1}(t)=A_{0}\exp(-a\times 2\pi f_{n}t)\times \sin(2\pi f_{n}\times \sqrt{1-a^{2}}t)\\ x_{2}(t)=A_{1}[\sin(2\pi f_{1}t)+\cos(2\pi f_{2}t)] \end{array}\right.$$

where *A*_0_ = 1 is intensity of fault impulse impact, *A*_1_ = 0.3 is intensity of systematic vibration signal, damping ratio *a* = 0.1, *f_n_* = 2000 represents the natural frequency of excited structure, the length of vibration signal *N* = 5120, the rotating frequencies are *f*_1_ = 120 Hz and *f*_2_ = 300 Hz, and the sampling frequency *f_s_* = 20 KHz. Experiments were carried out on a computer with Windows 10, quad-core processors at 2.9 GHz CPU, and 16 GB RAM. Figure 4 depicts the obtained synthetic simulation vibration signal.

**Figure 5:**
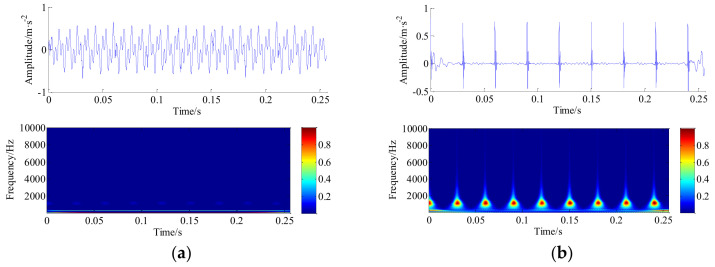
The decomposition results of synthetic simulation signal with TQWT-MCA method. (**a**) HRC and its wavelet time-frequency diagram; and (**b**) LRC and its wavelet time-frequency diagram.

Then, the TQWT-MCA method is introduced to process the raw simulated synthetic signal. The decomposition was obtained using the high-*Q* with parameters *Q*_2_ = 7, *r*_2_ = 3, *j*_2_ = 30 levels, and using the low-*Q* with parameters *Q*_1_ = 1, *r*_1_ = 3, *j*_1_ = 8 levels (as illustrated in Table 1). The algorithm is implemented for 100 iterations to minimize the objective function $F(w_{1},w_{2})$ in Equation (5). The parameters of the proposed algorithm are also listed in Table 1. The high-resonance and low-resonance components obtained by minimizing Equation (5) are illustrated in Figure 5a,b. From Figure 5, it should be noted that non-oscillatory behavior of the low-resonance component (LRC) and oscillatory behavior of the high-resonance component (HRC) can be reasonably described, which illustrate excellent separation results. 

**Figure 6:**
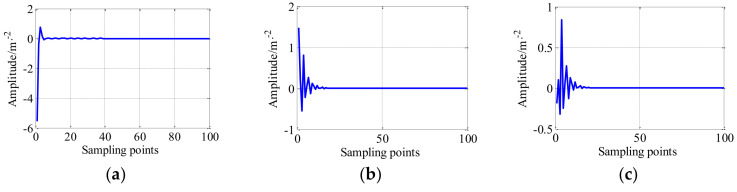
The time-domain waveform of (**a**) impulse-like impact atom; (**b**) step-like impact atom; and (**c**) impulse-step impact atom.

For the design of the dictionary atom, according to the range of amplitude of LRC generated by TQWT-MCA is [−0.5, 1], the parameters of the impulse-step impact dictionary atom are set as follows: the system damping constant *τ* is 0.001, peak value ratio *a* is 0.3, the system natural frequency *f_n_* = 10,000 Hz, the impulse-like response happened *u* is 0.005, the rotor speed rotation frequency *f_r_* is 800 rpm, and adjusting parameters $\eta_{1}=0.1$ and $\eta_{2}=0.1$. The time-domain waveforms of the impulse-like atom, step-like impact atom, and impulse-step-like impact atom are shown in Figure 6. As shown in Figure 6c, the range of the amplitude of the dictionary atom is [−0.5, 1].

The main evaluation purpose of the proposed method is that diagnosis task is first performed on the raw dataset, and then the same diagnosis task is performed on the recovered dataset, finally, the results of the two tasks are compared. If the results are equivalent or approximately equivalent, which means the recovered dataset has a high fidelity, and the diagnosis task will remain unaffected. Otherwise, if the results are far from each other, which means the recovered dataset is seriously distorted. 

**Figure 7:**
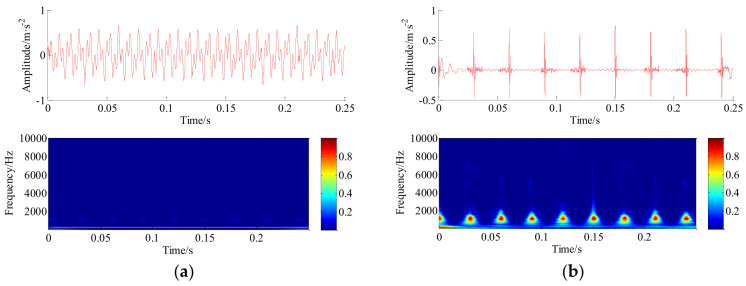
The reconstructed signals based on sparse Bayesian iteration framework. (**a**) High-frequency signal and its wavelet time-frequency diagram (from top to bottom); (**b**) low-frequency signal and its wavelet time-frequency diagram (from top to bottom).

**Figure 8:**
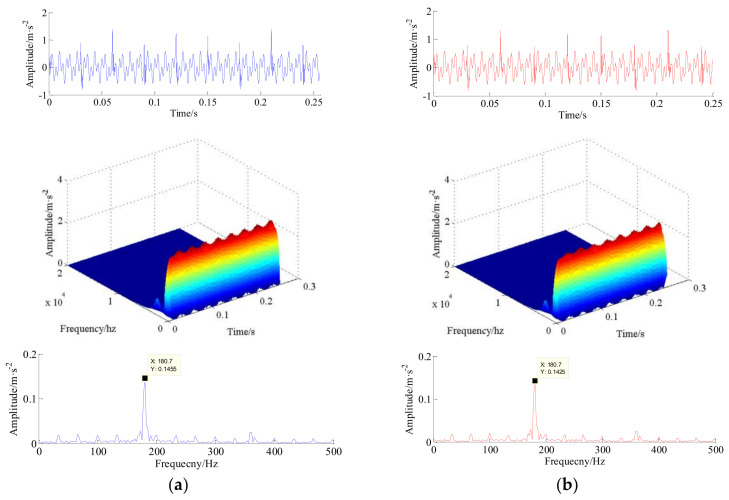
The raw simulated synthetic signal and the reconstructed synthetic signal. (**a**) the raw simulated synthetic signal, 3D-STFT time-frequency diagram and envelope spectrum (from top to bottom); (**b**) the reconstructed signal, 3D-STFT time-frequency diagram and envelope spectrum (from top to bottom).

Based on this point, the decomposed HRC and LRC are compressed and then recovered by sparse Bayesian iteration framework. The raw simulated synthetic signal, reconstructed signal of the HRC and LRC and their time-frequency diagrams are shown in Figure 7a,b, respectively. Taken summing, the reconstructed synthetic signal, and its 3D short-time Fourier transform (STFT) time-frequency diagram and envelope spectrum are shown in Figure 8b. In the experiment, the comparison results are evaluated using a time-frequency diagram because a small disturbance can lead to larger shadow in the time-frequency diagram. Moreover, it can be found that the peak point in envelope spectrum of original signal is (180.7, 0.1455), and the peak point in envelope spectrum of reconstructed signal is (180.7, 0.1425), thus the results indicate that our proposed algorithm is advantageous in this numerical simulation application. The practical applications of the proposed technique for multichannel vibration signals reconstruction and their fault diagnosis of the rotating machines will be investigated in the following section.

## 5. Experimental Case and Discussion

**Figure 9:**
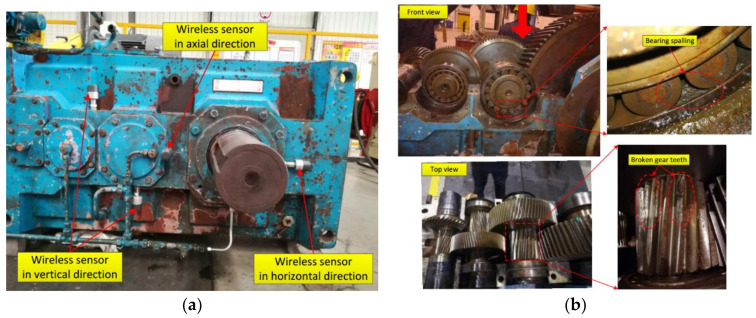
Experimental setup of gearbox with multi-fault. (**a**) Before dismantling; and (**b**) after dismantling.

To demonstrate the validity of the proposed approach for multiple-channel signals in engineering applications, the large reducer gearbox with multi-failure is implemented, the overall experimental setup is shown in Figure 9, Figure 9a is the experimental setup before dismantling and Figure 9b is the failure units after dismantling. As we can see from Figure 9b, the gears broken teeth might be caused by fatigue, and the spalling failures found in bearing outer race and bearing ball might be caused by harsh operating environment, such as high-temperature and humidity or lack of lubrication, etc. The experimental vibration acceleration data were collected from several accelerometers instrumented on bearing end bracket with eight channels. The geometrical parameters of the tested tapered bearing (FAG-32310-A) are listed in Table 2, and the transmission ratio and meshing frequency of the test gearbox are summarized in Table 3, respectively. The sampling frequency is 5120 Hz, the rotation frequency of the input shaft is 2114 rpm (i.e., 35.23 Hz), and sampling length is 16 s. In this experiment, after dismantling, the bearing spalling failure and the broken gear teeth are found at shaft IV, the fault frequency of the bearing outer race is 170 Hz, fault frequency of the bearing ball is 7.4 Hz and fault frequency of the broken gear is 21.607 Hz. 

**Figure 10:**
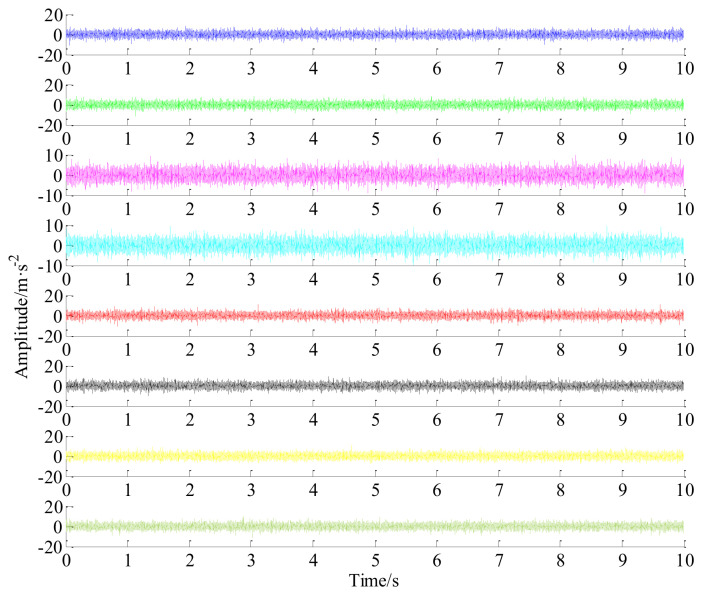
The raw vibration signal with eight channels.

In this experiment, a series of acceleration sensors, i.e., WD-ACWL500 wireless sensors (it obeys Zigbee wireless communication protocol), were used for signal acquisition. The vibration data were recorded from bearing bracket (see Figure 9a) of the shaft-IV using an eight-channel NI sampling system (the acquisition equipment was omitted in Figure 9), the raw vibration signal (51,200 sampling points are selected, i.e., 10 s) from channel #1 to channel #8 are displayed in Figure 10. The top row is channel 1#, the second row is channel #2 and the bottom is channel #8 accordingly. To clearly examine the data recovery quality, channel #1 and channel #8 are randomly chosen as the research objects. 

**Figure 11:**
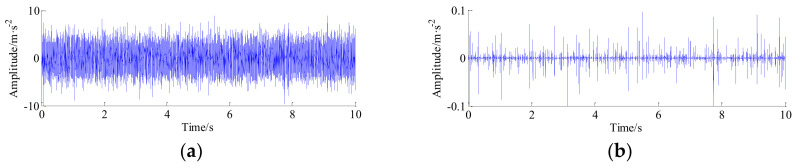
The decomposition results of raw signal of channel #1with TQWT-MCA method. (**a**) High-frequency signal; and (**b**) low-frequency signal.

Furthermore, time-domain signal is processed by the TQWT-MCA method. The decomposition parameters of the TQWT-MCA method are illustrated in Table 4. The decomposition results related to the high-resonance and low-resonance behavior are shown in Figure 11a,b, respectively. As can be seen from the Figure 11b, the periodic impulses related to the fault information can be peeled by the TQWT-MCA method from the raw vibration signal one by one, meanwhile, the noise has been also effectively suppressed.

**Figure 12:**
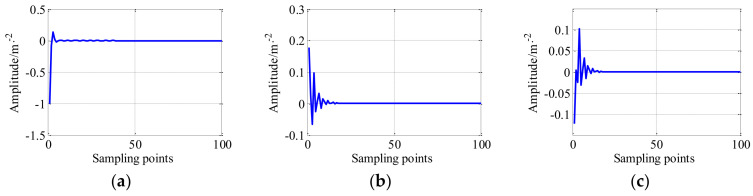
The time-domain waveform of (**a**) impulse-like impact atom; (**b**) step-like impact atom; and (**c**) impulse-step impact atom.

Before reconstruction, the dictionary atom is designed for dictionary learning, similar, according to the range of amplitude of LRC generated by TQWT-MCA is [−0.1, 0.1], the parameters of the impulse-step impact dictionary atom are set as follows: the system damping constant *τ* is 0.001, peak value ratio *a* is 0.3, the system natural frequency *fn* = 10,000 Hz, the impulse-like response happened *u* is 0.005, the rotor speed rotation frequency *fr* is 800 rpm, and adjusting parameters $\eta_{1}=0.018$ and $\eta_{2}=0.012$. The time-domain waveforms of the impulse-like atom, step-like impact atom, impulse-step-like impact atom are shown in Figure 12. As shown in Figure 12c, the range of the amplitude of the dictionary atom is [−0.1, 0.1]. 

**Figure 13:**
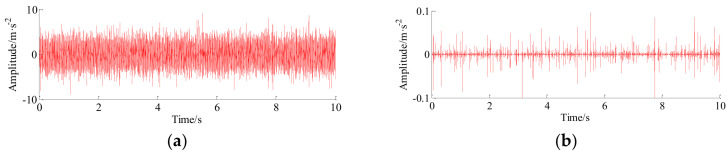
The reconstructed signals based on sparse Bayesian iteration framework. (**a**) reconstructed HRC; (**b**) reconstructed LRC.

**Figure 14:**
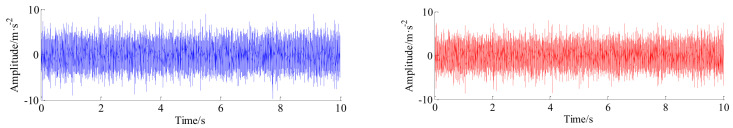

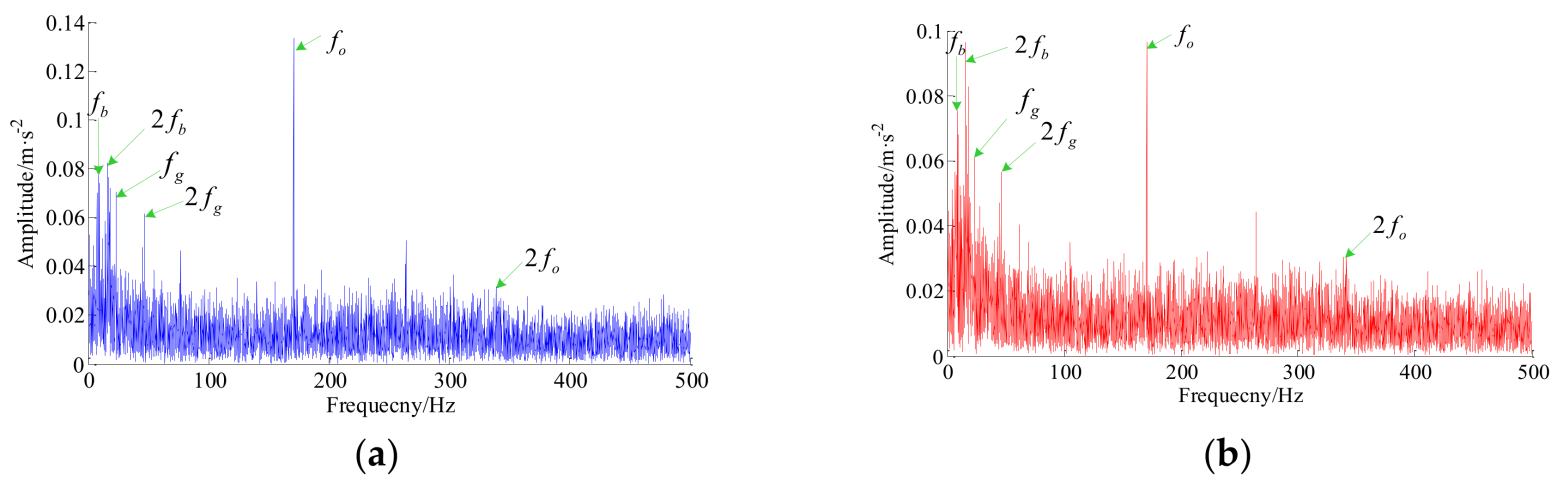
The raw signal of channel #1, reconstructed signal and their envelope spectrums. (**a**) The raw simulated synthetic signal and its envelope spectrum (from top to bottom); (**b**) the reconstructed signal and its envelope spectrum (from top to bottom).

The reconstructed signals of the HRC and LRC based on sparse Bayesian iteration framework are shown in Figure 13a,b, respectively. As shown in Figure 13b, the transient impulses are recovered and preserved well as indicated by impulses interval and impulses amplitude. Merge operations are executed using both two reconstructed signals, the reconstructed signal and its envelope spectrum are shown in Figure 14b. Figure 14a is the raw vibration signal and its envelope spectrum. Comparing these time-domain signals and envelope spectrums, it should be noted that, for the clear feature spectrum lines, the outer fault frequency (170.5 Hz) and its harmonic (341.7 Hz) can be detected by the proposed method. More importantly, for the weak feature spectrum lines, the broken gear fault (23.36 Hz) and its harmonics (46.72 Hz and 69.22 Hz, etc.), the bearing element fault (7.344 Hz) and its harmonics (15.86 Hz) can be also distinguished in the envelope spectrum of the reconstructed signal, which demonstrate that vibration signal in channel #1 is effectively recovered. 

**Figure 15:**
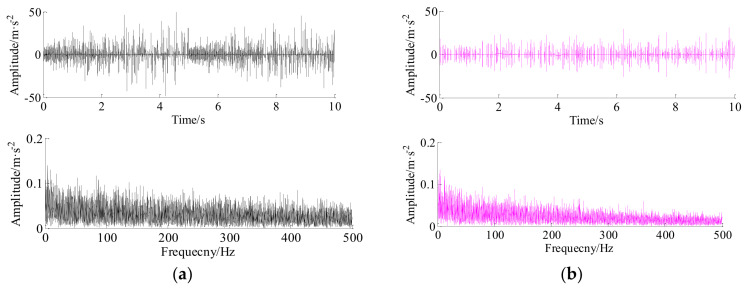

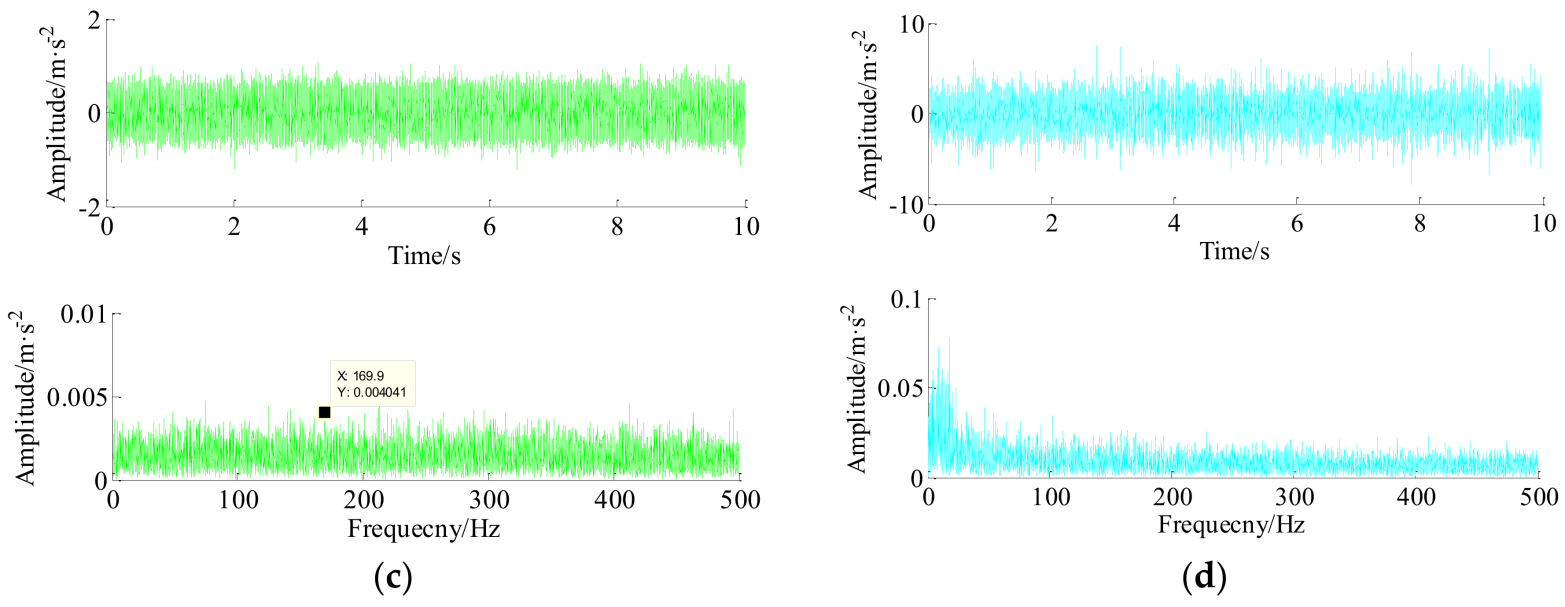
The reconstructed signals and their envelope spectrums with benchmark methods. (**a**) Orthogonal matching pursuit (OMP); (**b**) Lq-norm (q = 0.5) method; (**c**) L1-norm; and (**d**) the spatiotemporal sparse Bayesian learning (SSBL) method.

**Figure 16:**
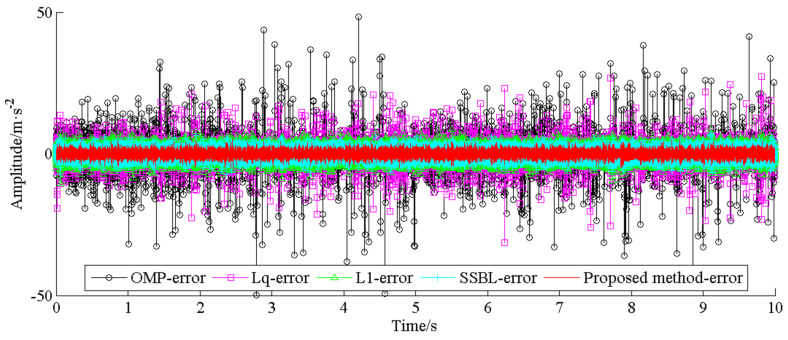
Error contrast waveform of proposed method and four benchmark methods.

As the benchmark approaches for signal reconstruction and fault detection, the signal of channel #1 is respectively compressed and then recovered by orthogonal matching pursuit (OMP), convex L1-norm, and non-convex Lq-norm (q = 0.5) methods [16] and spatiotemporal sparse Bayesian learning (SSBL) [22], and the reconstructed signals and their frequency spectrums are respectively shown in Figure 15. As shown in Figure 15c, only the spectrum peak at 169.9 Hz that is consistent with the fault frequency of the bearing outer race can be identified by the convex L1-norm, other fault information cannot be found in other frequency spectrums, e.g., OMP and non-convex Lq-norm (q = 0.5). This is because the objective cost function in Equation (10) is convex when the L1-norm was employed. However, the objective function in Equation (10) will not be convex when the non-convex Lq-norm (q = 0.5) is employed and its solution may fall into the local optimum. From fig 15d, it is note that the fault frequencies of bearing ball and broken gear can be identify (the amplitudes are not obvious), but the fault information of bearing outer race cannot be detected in the envelope spectrum. The results indicating that the fault impulses of bearing outer race cannot be recovered well during signals reconstruction due to the physical structure of fault impulses does not match well with discrete Cosine transform (DCT). Moreover, Figure 16 gives the comparison amplitude error between the original vibration signal and the final reconstructed signal with the different color lines, respectively. 

Additionally, the running time for the channel #1 signal with different algorithms are listed in Table 5. The running time of the proposed method is greatly reduced as compared to traditional CS approaches. Overall, it can be observed that the reconstructed result generated by the proposed method is well matched with the raw signal in time domain, which also proves the effectiveness of the proposed reconstruction method.

**Figure 17:**
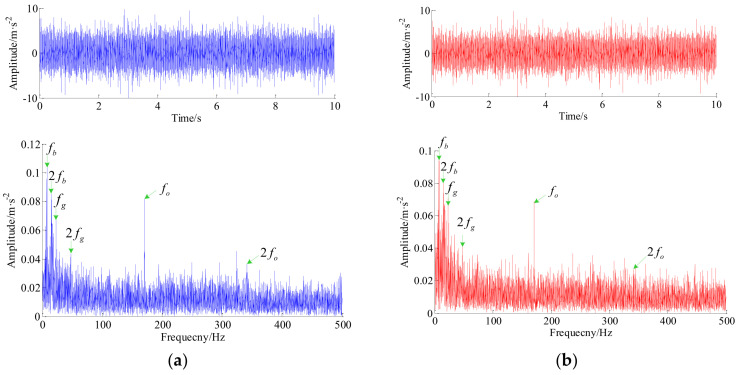
The raw signal of channel #8, reconstructed signal and their envelope spectrums. (**a**) The raw vibration signal and its envelope spectrum (from top to bottom); and (**b**) the reconstructed signal and its envelope spectrum (from top to bottom).

Additionally, we continue to analyze the raw vibration signal from channel #8. The waveform of the original signal and its envelope spectrum, the reconstructed signal generated by the proposed algorithm and its envelope spectrum are shown in Figure 17a,b, respectively. Apparently, the clear bearing outer race fault frequency at 170.7 Hz and its harmonics at 341.3 Hz, the bearing ball fault frequency at 7.344 Hz and its harmonics at 15.0 Hz, and the gear fault frequency at 23.36 Hz and its harmonics at 47.42 Hz are detected in envelope spectrum.

In conclusion, the above result demonstrates that the proposed algorithm can recover the multichannel signals with high accuracy, and the weak fault information can be immaculately preserved.

## 6. Conclusions

In this paper, to relieve pressure from data storage and remote transmission with data increasing daily, a novel multichannel signal reconstruction method based on TQWT-MCA and a sparse Bayesian iteration algorithm, combined with step-impulse dictionary, is proposed for the PHM of rotating machinery. The results obtained from this research are as follows:

(1) The raw vibration signal is decomposed into a high-resonance component and low-resonance component, in order to avoid the distortion and aliasing of the periodical impulses when SBL is implemented, and also the periodical impulses caused by the localized fault are preserved. Meanwhile, the dictionary atom is designed to match the physical structure of periodical impulses.
(2) In contrast to existing compressed sensing algorithms, the proposed method not only exploits correlation structures within a single channel signal, but also exploits multiple-channel correlation, which means the spatiotemporal relationships among the signals from different channels are considered via the updating and learning rule of the matrix *A* and matrix *B*. The HRC and LRC can be recovered with high accuracy based on the Bayesian iteration algorithm combined with step-impulse dictionary.
(3) Due to it has much better recovery performance than state-of-the-art algorithms, thus, the weak fault information can be immaculately preserved. Meanwhile, the proposed method may relieve the pressure from long-term prognostic and health management in terms of data storage and remote transmission.

Although the proposed method improves the reconstruction quality significantly, it still needs future improvements, as shown in Table 5, the running time of OMP for channel 1# is 98.28 s, the Lq-norm is 952.39 s, the L1-norm is 228.76 s, it is noted that the running time of proposed method is faster than the above CS methods, but slower than the spatiotemporal sparse Bayesian learning (SSBL) (i.e., 3.02 s), therefore, compare with the SSBL method, the complexity level and computational time of the proposed approach is rather high due to dictionary training and its iterations operation. It is suggested that faster calculation methods will be explored in future studies.

## Figures and Tables

**Table 1:** Algorithm parameters used in the numerical simulation.

**High-*Q*_2_**	**Low-*Q*_1_**	**Redundancy Rate *r*_2_**	**Redundancy Rate *r*_1_**	**Decomposition Levels *j*_2_**
7	1	3	3	30
**Decomposition Levels *j*_1_**	**Iteration Times**	**Regularization Parameters**	**Regularization Parameters**	
8	100	$\lambda_{1}=0.01$	$\lambda_{2}=0.01$	

**Table 2:** The geometrical parameters of the tested tapered rolling bearing.

Bearing Type	Fault Type	Number of Balls	Inner Diameter	Outer Diameter	Outer Race Fault Frequency	Ball Fault Frequency
FAG-32310-A	Outer race	16	50 mm	110 mm	170 Hz	7.4 Hz

**Table 3:** The transmission ratio and meshing frequency of the test gearbox.

Axis	Bull Gear	Pinion Gear	Speed Ratio	Rotational Frequency	Meshing Frequency
**Shaft-I**	-	19	-	35.233 Hz	669.433 Hz
**Shaft-II**	34	20	0.56	19.689 Hz	393.784 Hz
**Shaft-III**	81	20	0.25	4.862 Hz	97.231 Hz
**Shaft-IV**	81	18	0.25	1.200 Hz	21.607 Hz
**Shaft-V**	88	-	0.20	0.246 Hz	

**Table 4:** Algorithm parameters used in the practical signal of channel #1.

**High-*Q*_2_**	**Low-*Q*_1_**	**Redundancy Rate *r*_2_**	**Redundancy Rate *r*_1_**	**Decomposition Levels *j*_2_**
7	1	3	3	30
**Decomposition Levels *j*_1_**	**Iteration Times**	**Regularization Parameters**	**Regularization Parameters**	
8	100	$\lambda_{1}=0.01$	$\lambda_{2}=0.01$	

**Table 5:** Running time for channel #1 signals with different algorithms.

OMP (s)	Lq-Norm (s)	L1-Norm (s)	SSBL (s)	Proposed Method (s)
98.28	952.39	228.76	3.02 (24.19/8)	9.08 (72.67/8)
